# Recent Progress of Ferroelectric-Gate Field-Effect Transistors and Applications to Nonvolatile Logic and FeNAND Flash Memory

**DOI:** 10.3390/ma3114950

**Published:** 2010-11-18

**Authors:** Shigeki Sakai, Mitsue Takahashi

**Affiliations:** National Institute of Advanced Industrial Science and Technology / Central 2, 1-1-1 Umezono, Tsukuba, Ibaraki 305-8568, Japan; E-Mail: mitsue-takahashi@aist.go.jp

**Keywords:** FeFET, semiconductor memory, nonvolatile memory, nonvolatile logic

## Abstract

We have investigated ferroelectric-gate field-effect transistors (FeFETs) with Pt/SrBi_2_Ta_2_O_9_/(HfO_2_)_x_(Al_2_O_3_)_1−x_ (Hf-Al-O) and Pt/SrBi_2_Ta_2_O_9_/HfO_2_ gate stacks. The fabricated FeFETs have excellent data retention characteristics: The drain current ratio between the on- and off-states of a FeFET was more than 2 × 10^6^ after 12 days, and the decreasing rate of this ratio was so small that the extrapolated drain current ratio after 10 years is larger than 1 × 10^5^. A fabricated self-aligned gate Pt/SrBi_2_Ta_2_O_9_/Hf-Al-O/Si FET revealed a sufficiently large drain current ratio of 2.4 × 10^5^ after 33.5 day, which is 6.5 × 10^4^ after 10 years by extrapolation. The developed FeFETs also revealed stable retention characteristics at an elevated temperature up to 120 °C and had small transistor threshold voltage (*V*_th_) distribution. The *V*_th_ can be adjusted by controlling channel impurity densities for both *n*-channel and *p*-channel FeFETs. These performances are now suitable to integrated circuit application with nonvolatile functions. Fundamental properties for the applications to ferroelectric-CMOS nonvolatile logic-circuits and to ferroelectric-NAND flash memories are demonstrated.

## 1. Introduction

As a nonvolatile memory, ferroelectric-gate field-effect-transistors (FeFETs) have many advantages in high-density integration, low power dissipation, non-destructive readout operation, and good scalability [1]. A variety of FeFETs had been investigated over the past decades [2,3,4,5,6,7,8,9,10]. However, despite much effort by a lot of research groups, data retention time of the FeFETs has been short. In order to explain the cause of this short data retention, the effects of depolarization field [11] and unsaturated polarizations [12] in ferroelectric layers have been discussed.

A promising gate-material combination of metal/ferroelectric/insulator/semiconductor (MFIS) and a good process for FeFETs having long data retention were found by the author (S.S.) in 2002 [13,14,15,16,17,18]. Since then, FeFETs became a real candidate for practical nonvolatile memories. We have continuously studied not only further technological development of FeFETs but also FeFET applications to integrated circuits. We are now investigating two kinds of applications of the FeFETs, which are FeCMOS nonvolatile logic circuits and FeNAND flash memories. Note that FeFETs based on different materials and on different types have been investigated during the last decade, which are listed only partially in [19,20,21,22,23,24,25,26].

In this paper, we will first describe our FeFET development, and second, we will show recent results of the FeFET applications to FeCMOS and FeNAND flash memories.

## 2. Progress of Ferroelectric-Gate Field-Effect-Transistors

**Figure 1:**
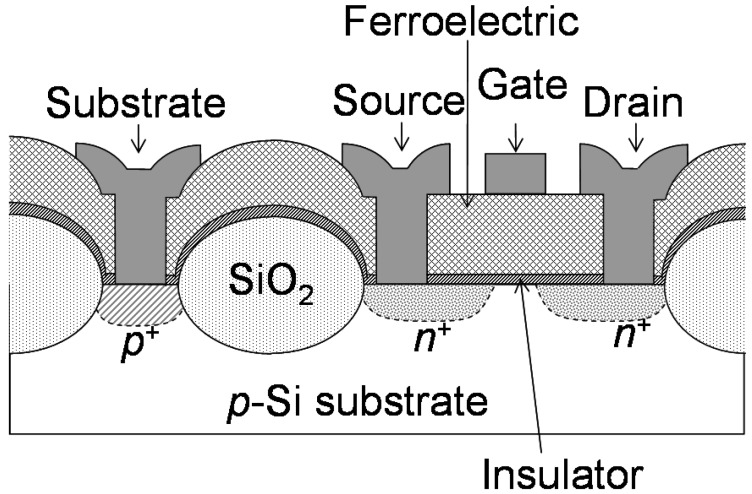
Schematic cross section of a fabricated FeFET. Gate, ferroelectric and insulator are made of Pt, SBT and Hf-Al-O, respectively. Reprinted from [17] with permission of the Japan Society of Applied Physics.

**Figure 2:**
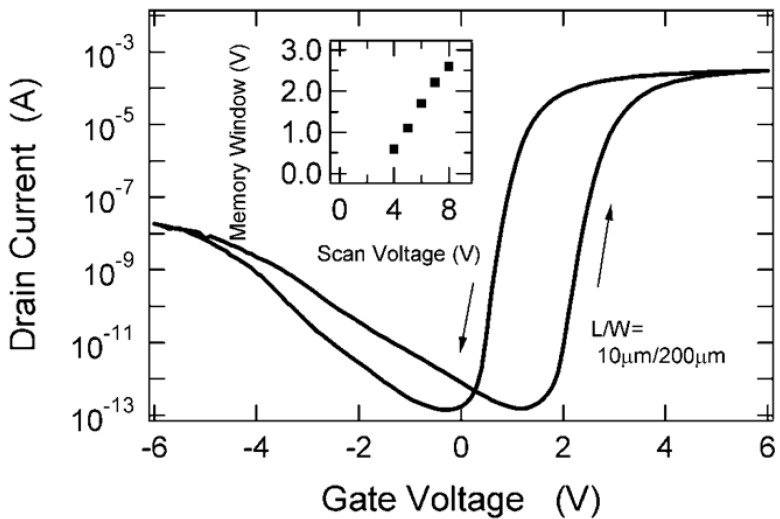
Drain current-gate voltage curves of an *n*-channel Pt/SBT/Hf-Al-O/Si FeFET at *V*_g_ = ±6.0 V. The inset was made by measuring the curves at *V*_g_ = ±4.0, ±5.0, ±6.0, ±7.0 and ±8.0 V. Scan voltage is each gate-voltage amplitude applied to the FeFET. The almost linearly increasing memory window shown in the inset indicates that ferroelectric polarization in the FeFET is not saturated even at *V*_g_ = ±8.0 V. Reproduced from [15] with permission of IEEE.

The promising material combination of the MFIS gate stack discovered in 2002 was Pt/SrBi_2_Ta_2_O_9_/(HfO_2_)_x_(Al_2_O_3_)_1−x_/Si. Hereafter, SrBi_2_Ta_2_O_9_ and (HfO_2_)_x_(Al_2_O_3_)_1−x_ are abbreviated as SBT and Hf-Al-O, respectively. A schematic cross section is shown in Figure 1. The *I*_d_ *vs.* gate voltage (*V*_g_) characteristic for an *n*-channel MFIS FET (x = 0.75) is shown in Figure 2 [15]. When the applied *V*_g_ was varied from −6.0 V to +6.0 V, a hysteresis loop with a wide memory window of 1.6 V and a large *I*_d,on_/*I*_d,off_ ratio over 10^7^ at *V*_g_ = 1.7 V was obtained due to the ferroelectricity. The *I*_d_ increase at negative *V*_g_ in Figure 2 is not due to a gate leakage current, but due to a current between the *p*-type substrate and the *n*^+^-drain. This *I*_d_ increase was closely correlated to the overlap length of the drain and gate. When this overlap length was shortened, the drain current increase at the negative gate voltage was lowered. Thus, the increased current at the negative gate voltages is a gate-induced leakage current (GIDL) between the drain bulk (*n*^+^) and the drain-surface inversion (*p*) layer under the gate area [15].

**Figure 3:**
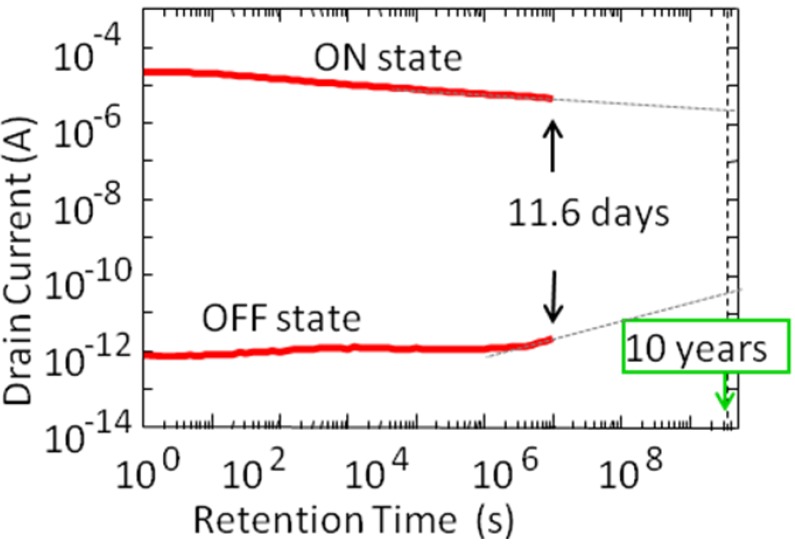
Drain current retention of a Pt/SBT/Hf-Al-O/Si FeFET. Potential 10 year retention is indicated by the extension lines on the measured retention curves. Modified from [15].

Figure 3 shows *I*_d_ data retention characteristics of the MFIS FET [13,14,15,16,17,18]. During the *I*_d,on_ retention measurement, *V*_g_ was kept at a bias gate voltage *V*_keep_ = 1.7 V after *V*_g_ = +6.0 V was applied to polarize the ferroelectric SBT. For the *I*_d,off_ retention measurement, *V*_g_ was kept at the same bias gate voltage, *V*_keep_ = 1.7 V, after *V*_g_ = −6.0 V was applied. Both the *I*_d,on_ and *I*_d,off_ retention characteristics were measured up to 10^6^ s (12 days). The *I*_d,on_/*I*_d,off_ ratio was about 10^7^ immediately after data writing and still larger than 10^6^ after 12 days. A plot of the memory window *vs.* the applied *V*_g_ amplitude or scan voltage is shown in the inset of Figure 2. The inset indicates that the ferroelectric polarization is not saturated yet at a scan voltage of 8.0 V. This concludes that the usage of saturated polarization is not a necessary condition to get FeFETs with long retention. The fact that a FeFET with long retention was achieved means that nonvolatile FeFETs can work even under the presence of the depolarization field in the MFIS gate stack.

**Figure 4:**
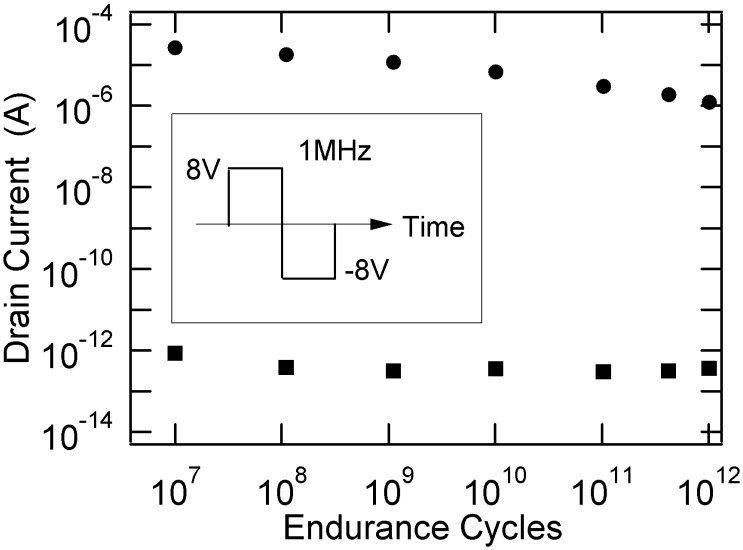
Pulse endurance property of a Pt/SBT/Hf-Al-O/Si FeFET. The inset shows a cycle of the gate voltages which were periodically applied. Reprinted from [15] with permission of IEEE.

Endurance tests were also performed [15]. A cycle of the endurance pulse is shown in the inset of Figure 4. The *I*_d,on_ and *I*_d,off_ were measured at *V*_keep_ = 2.0 V after a large number of the endurance pulse cycles were applied to the FET gate electrode. As shown in Figure 4, there was no serious deterioration until 10^12^ cycles. Even after 10^12^ cycles, the *I*_d,on_/*I*_d,off_ ratio was more than 10^6^.

**Figure 5:**
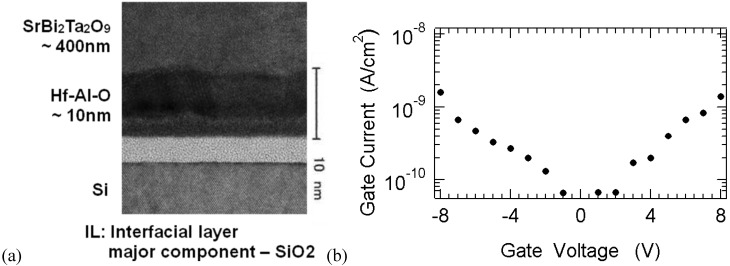
(a) Cross-sectional view of a gate by TEM, reprinted from reference [17] with permission of the Japan Society of Applied Physics, and (b) a gate-leakage property of Pt/SBT/Hf-Al-O/Si FeFETs, reprinted from reference [15] with permission of IEEE.

The fabrication process is typically as follows: On a substrate with the source- and drain-regions, an Hf-Al-O or HfO_2_ buffer insulating layer was deposited in 13 Pa N_2_-ambient by a pulsed-laser deposition (PLD) technique. The substrate temperature during the deposition was 200 °C. The SBT layer was successively deposited by PLD in 13 Pa O_2_-ambient at the substrate temperature of 400 °C. The gate metal Pt was electron-beam evaporated. In order to crystallize the SBT layer and to bring out the ferroelectric properties in this layer, the sample was annealed at 800 °C in O_2_ for 1 h. Key points for Pt/SBT/Hf-Al-O/Si long-retention FeFETs found in 2002 are summarized as follows: Among MFS, MFIS and MFMIS gate structures, the author chose the MFIS as the most promising structure. As a lower dielectric constant ferroelectric SBT was selected. SBT needs rather high temperature annealing, and in fact a high temperature process of 800 °C and 1h was used to realize the inherent high ferroelectricity of SBT. There are a lot of requirements for the insulating layer (*I*) in MFIS. It should be a good oxygen diffusion barrier to reduce SiO_2_-like interfacial layer formation on the Si surface, and should be a high-*k* material to get a large voltage across *F* layer. It should also be strong for SBT annealing around 800 °C and should have chemically stable interface between *F* and *I*. Further, it should have good interface between *I*/Si as the channel of the memory transistors, and should be dense to have small leakage currents. These must have been verified by making MFIS FETs ***actually.*** The Hf-Al-O chosen as the layer met all the requirements. The interfacial layer was formed but the thickness was acceptable for device operations. (A cross-sectional transmission electron microscopy (TEM) photo is shown in Figure 5(a)) The Hf-Al-O shows dense amorphous properties. The *F*/*I* interface was chemically stable. The gate leakage current was suppressed to the order of or less than 10^−9^ A/cm^2^ as shown in Figure 5(b). The composition ratio x = 1 of the insulator (HfO_2_)_x_(Al_2_O_3_)_1-x_ is HfO_2_. Pt/SBT/HfO_2_/Si FeFETs have also shown low gate-leakage current and excellent retention characteristics [13,16,27].

**Figure 6:**
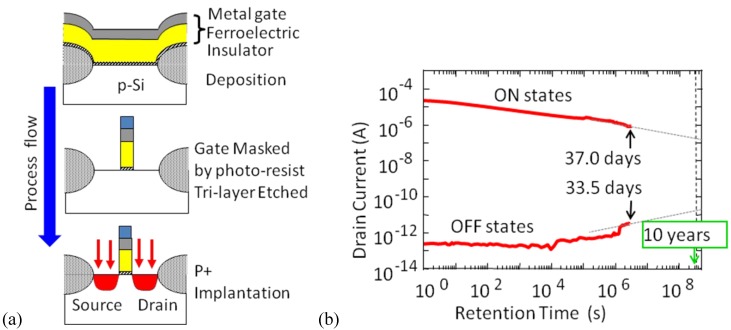
(a) Self-aligned-gate process for fabricating FeFETs; (b) Drain current retention of a self-aligned-gate Pt/SBT/Hf-Al-O/Si FeFET with *L* = 2µm. Respective curves for on- and off-states were measured over one month. Thicknesses of the SBT and Hf-Al-O were 420 nm and 12 nm. Modified from [28].

In 2005 we produced self-aligned-gate FeFETs (Figure 6(a)) with gate length *L* = 2µm and succeeded in measuring 33.5 day-long data retentions (Figure 6(b)) [28]. By extrapolating from the obtained curves for on- and off-retention in Figure 6, *I*_d_ on/off ratio over 10^4^ times is expected at 10 years after writing the data. There is also a report of Pt/SBT/HfO_2_/Si FeFET with one month retention [29]. Since the FeFET retention was no longer a crucial problem to be solved, we investigated other reliabilities of device performance at elevated temperatures [30,31], threshold-voltage (*V*_th_) distribution [32], and *V*_th_ adjustment [33].

**Figure 7:**
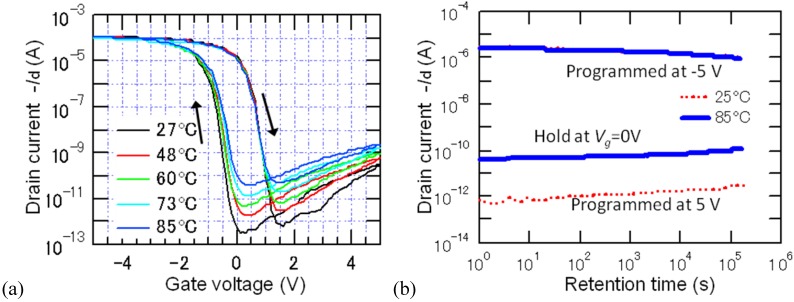

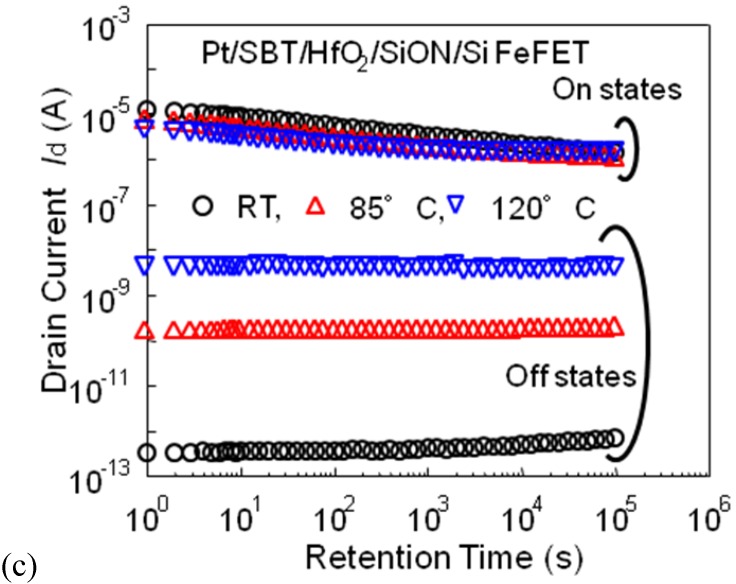
Electrical properties of FeFETs measured at elevated temperatures. (a) Drain current-gate voltage curves, and (b) the drain current retentions of a *p*-channel Pt/SBT/Hf-Al-O/Si FeFET. Thicknesses of the SBT and Hf-Al-O were 600 nm and 7 nm. Reprinted from [30] with permission of the American Institute of Physics. Good stability of FeFET data retention even at 120 °C was also supported by measuring the retentions of an *n*-channel Pt/SBT/HfO_2_/SiON/Si FeFET (c). Thicknesses of the SBT and HfO_2_ were 450 nm and 6 nm. Reprinted from [31] with permission of IOP Publishing Ltd.

Figures 7 (a) and (b) showed very stable retention performance of a *p*-channel Pt/SBT/Hf-Al-O/Si FeFET at elevated temperatures up to 85 °C [30]. We also succeeded to obtain good retentions of an *n*-channel Pt/SBT/HfO_2_/SiON/Si FeFET at 120 °C (Figure 7(c)) [31]. Figure 7(a) indicated that rising current levels of off-state retention curves shown in Figure 7(b) and (c), as the measurement temperature was increased, is due to temperature dependence of source-drain conduction in the FeFETs at their off states.

**Figure 8:**
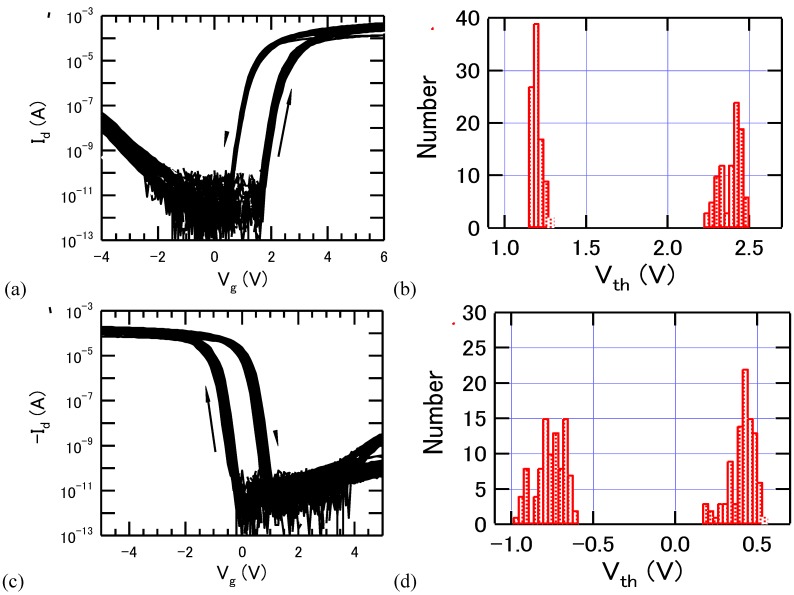
(a) Drain current-gate voltage curves of 94 *n*-channel Pt/SBT/Hf-Al-O/Si FeFETs and (b) *V*_th_ distribution of the 94 *n*-channel FeFETs; (c) Drain current-gate voltage curves of 95 *p*-channel Pt/SBT/Hf-Al-O/Si FeFETs and (d) *V*_th_ distribution of the 95 *p*-channel FeFETs. Thicknesses of the SBT and Hf-Al-O were 400 nm and 7 nm. Reprinted from reference [32] with permission of IOP Publishing Ltd.

As shown in Figures 8, statistical distribution of the threshold voltage *V_th_* for more than 90 Pt/SBT/Hf-Al-O/Si FeFETs distributed on an area of about 10 × 11 mm^2^ was estimated for both *p*- and *n*-channel devices. The average memory window was nearly 1.2 V at the sweep voltage amplitude of 5 V. The standard deviations of *V_th_* were about 7–8% and 3–5% of the memory window for the *n*-channel and *p*-channel FeFETs, respectively. These results indicate that the FeFET technology is up to a promising level for demonstrating an integrated circuit.

With regard to the downsizing of FeFETs, we have very recently fabricated FeFETs with *L* = 0.56 µm by using 200 nm-thick SBT [34]. As well as the early FeFETs introduced above in this chapter, the 0.56 µm FeFET showed good characteristics such as a memory window of 0.93 V, data retention measured over one day, and 10^8^ cycle endurance.

## 3. FeCMOS Nonvolatile Logic Circuits

Nonvolatile logic circuits with nonvolatile-memory function have attracted much interest for application to next-generation mobile devices with high-speed and low-power consumption [35,36]. We have proposed FeCMOS circuits which are complementary metal-oxide-semiconductor (CMOS) circuits composed of FeFETs instead of conventional MOS FETs [37,38]. The FeFETs have both the *n*-channel-type and *p*-channel-type as conventional MOS FETs have. A FeFET works as a logic transistor or a conventional MOS transistor when a voltage difference between the gate and the substrate (*V*_gsub_) is small enough to show negligibly narrow memory windows in *I*_d_-*V*_g_ curves. On the other hand, the FeFET works as a nonvolatile memory transistor when the *V*_gsub_ is large enough to show wide memory windows in the *I*_d_-*V*_g_ curves.

**Figure 9:**
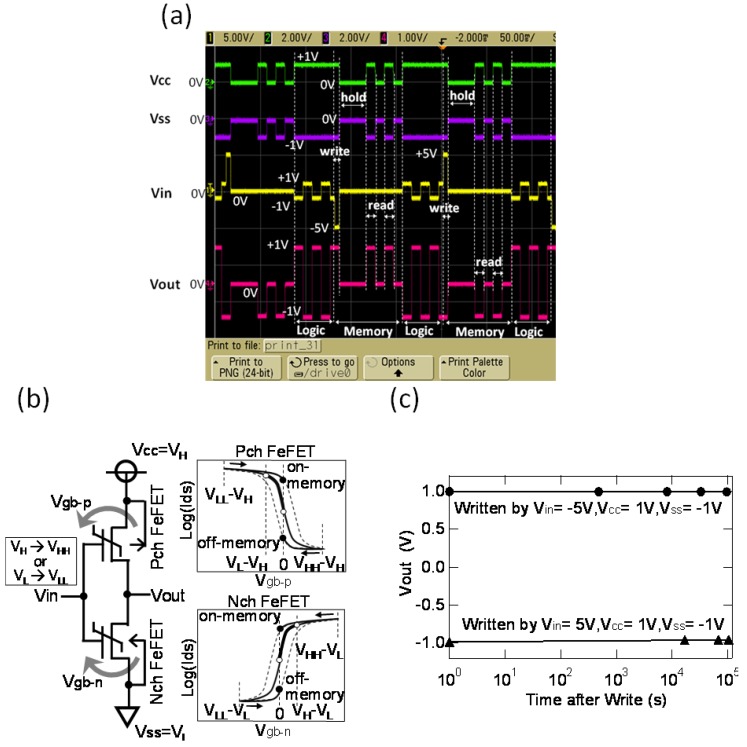
(a) Schema explaining nonvolatile logic operation of the FeCMOS inverter; (b) Demonstrations of operational switching between logic and memory mode of the FeCMOS inverte; (c) *V*_out_-data retention with nondestructive readout. Reproduced from [37] with permission of the Institution of Engineering and Technology.

Principles of logic-and-memory function switching are demonstrated in Figure 9 which show a single-stage FeFET inverter with the gate-signal amplitude directly changed [37]. At the logic operation, *V*cc is set to *V*_H_ and *V*ss is set to *V*_L_. *V*_H_ or *V*_L_ is given to the gate (*V*_in_) correspondingly, *V*_L_ or *V*_H_ appear to the output terminal (*V*_out_). In this normal inverter operation, the logic swing *V*_H_-*V*_L_ is so small that the *I*_d_-*V*_g_ curves both for *p*- and *n*-channel FeFETs are almost non-hysteretic (Figure 9(a)). At the memory operation, it consists of Write-, Sleep- and Read-modes. In order to write logic data at the input *V*_in_, *V*_in_ is increased to *V*_HH_ (*V*_HH_ >> *V*_H_) if *V*_in_ = *V*_H_, and is decreased to *V*_LL_ (*V*_LL_ << *V*_L_) if *V*_in_ = *V*_L_. Then, all supplied voltages are reduced to zero, and the circuit goes into Sleep mode. At the Read, correct information is readout by supplying voltages again to the power supply terminals (*V*cc = *V*_H_ and *V*ss = *V*_L_). This operation is non-destructive as shown in Figure 9(b). The long data retention was confirmed at the measurement of up to 1.2 days (Figure 9(c)).

**Figure 10:**
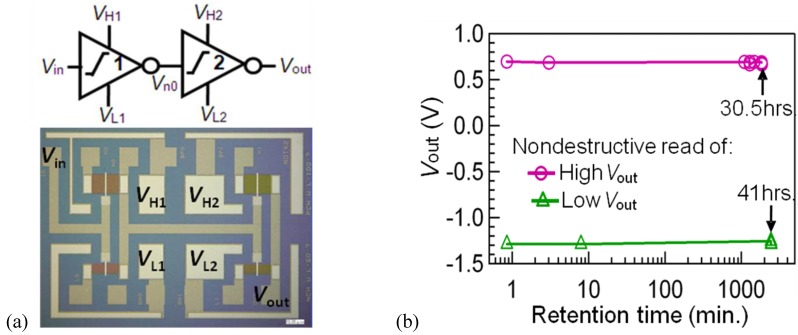
(a) Microscopic photo and (b) measured retention with nondestructive readout of double-stage FeCMOS inverter circuit. Reproduced from [38] with permission of IEICE.

In a practical usage, we do not know the input *V*_in_ status, *V*_H_ or *V*_L,_ at the write timing, but *V*_HH_ or *V*_LL_ has to be correctly given. A double-stage FeCMOS inverter circuit (Figure 10(a)) is the simplest circuit for this purpose. For data writing, the supplied voltages of the first stage were enhanced to *V*_H1_ = *V*_H_ + δ*V*_H_ and *V*_L1_ = *V*_L_ − δ*V*_L_. After that, the circuit went in the sleep mode by reducing all supply voltages to zero. Nondestructive data readout was confirmed by supplying again the voltages, *V*_H_ and *V*_L,_ only to the power supply terminals of the second stage. Data retentions of the circuit logic outputs (*V*_out_s) over 30.5 h were measured as shown in Figure 10(b) [38].

## 4. FeNAND Flash Memory

**Figure 11:**
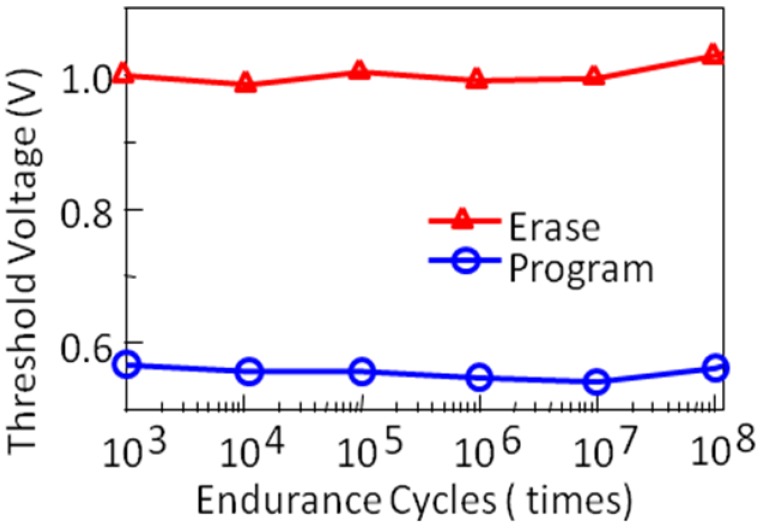
Program/erase endurance characteristics up to 10^8^ cycles of a FeNAND memory cell. Reproduced from [41] with permission of IEEE.

**Figure 12:**
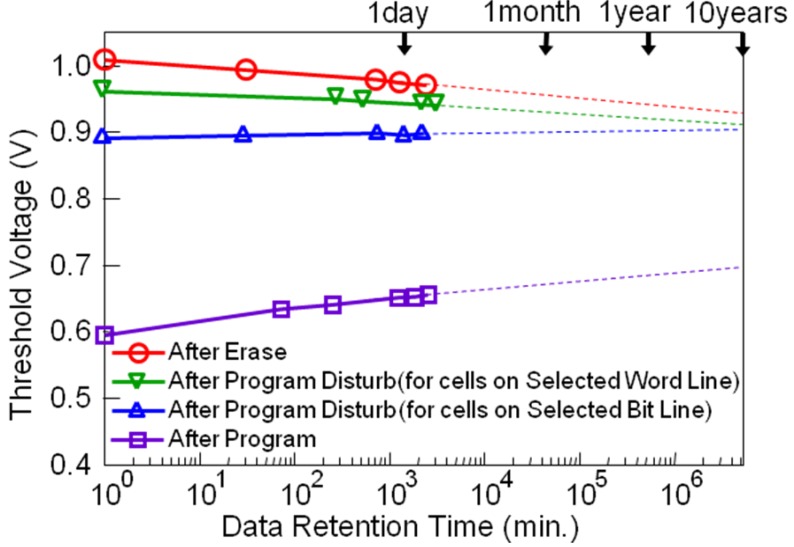
Data retention after the program, erase, *V*_pgm_ disturb and *V*_pass_ disturbs. Reproduced from [41] with permission of IEEE.

NAND flash memories are now popular high-density nonvolatile memories [39,40]. Conventional NAND flash memories are constructed by floating-gate (FG) type MOSFET memory cells. The mechanism of program and erase (P/E) operations is electron tunneling through a thin insulator between the FG and semiconductor channel. Program voltage for the FG-NAND cells is around 20 V and P/E cycle endurance is about 10^4^ times. As a solution to the problems of high operation voltage and low P/E endurance, we have proposed FeNAND flash memory using FeFETs instead of the FG-MOSFETs as memory cells. The FeNAND will have program voltages around 6 V and more than 10^8^ times P/E endurance [41]. The FeNAND is scalable by 4F^2^ rule (F: the feature size) as well as the FG-NAND. Capacitance-coupling noise problem to the adjacent cells can be expected to be small due to the very high permittivity of the ferroelectric even when they are downsized to 10–20 nm in the future. We investigated single-cell performance of the FeNAND. P/E endurance (Figure 11) and data retention after program, erase, *V*_pgm_ disturb and *V*_pass_ disturbs (Figure 12) were measured [41]. Ten year-long retention can be expected as suggested in Figure 12.

**Figure 13:**
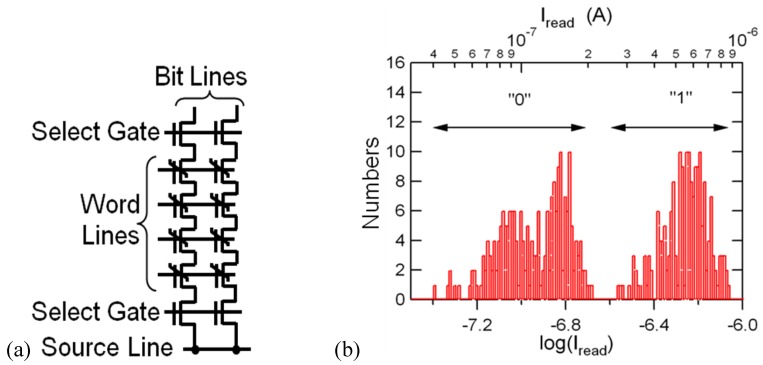
(a) Equivalent circuit of a 4 × 2 FeNAND memory cell array and (b) distribution of accumulated bit-line currents of 51 programmed patterns, which were measured every time after an erase-and-program cycle. Reproduced from [42] with permission of IOP Publishing Ltd.

Operations of arrayed ferroelectric (Fe-) NAND flash memory cells: erase, program and read, were demonstrated for the first time using a small cell array of four word lines by two NAND strings (Figure 13(a)). The memory cells and select-gate transistors were all *n*-channel Pt/SBT/Hf-Al-O/Si FeFETs. The erase was performed by applying 10 µs-wide 7 V pulses to *n*- and *p*-wells. The program was performed by applying 10 µs-wide 7 V pulses to selected word lines. Accumulated read currents of 51 programmed patterns in the FeNAND flash memory cell array successfully showed distribution of the two distinguishable “0” and “1” states [42] (Figure 13(b)). Retention times of bit-line currents were obtained over 33 hours for both the “0” and “1” states in a program pattern.

## 5. Conclusions

The developed Pt/SBT/Hf-Al-O/Si FeFETs have excellent long retention not only at room temperature but also at the elevated temperature of 120 °C. The FeFET threshold voltage positions could be well controlled and their distributions were small. Nonvolatile logic (FeCMOS) and NAND flash memory (FeNAND) are promising applications, and their fundamental operations were demonstrated by using the FeFET technology that we developed.
